# Gd‐Mediated Spin‐State Modulation of Co Sites Enhances Oxygen Electrocatalysis in Zn‐Air Battery

**DOI:** 10.1002/advs.78659

**Published:** 2026-11-05

**Authors:** Yijie Shen, Yu Zhu, Tianrui Zhao, Yiwen An, Xuan Wang, Meng Li, Dongmei Sun, Linda Zhang, Hao Li, Yawen Tang, Gengtao Fu

**Affiliations:** ^1^ Jiangsu Key Laboratory of Long‐duration Energy Storage Battery Technology Jiangsu Key Laboratory of Micro Nano Sensing and Separation Science for Analytical Chemistry School of Chemistry and Materials Science Nanjing Normal University Nanjing China; ^2^ Advanced Institute for Materials Research (WPI‐AIMR) Tohoku University Sendai Japan

**Keywords:** Co‐N‐C, Gd, oxygen reduction reaction, spin‐state modulation, Zn‐air battery

## Abstract

Co‐based catalysts are promising alternatives to precious‐metal catalysts towards oxygen reduction reaction (ORR), yet the limited electronic flexibility of Co sites constrains the adsorption and conversion of oxygen‐containing intermediates. Herein, we propose a Gd‐mediated spin‐state modulation strategy to enhance electrocatalytic ORR performance of Co sites. The incorporation of Gd into Co sites is realized by zeolitic imidazolate framework‐8, forming a Gd‐modified Co─N─C catalyst (GdCo/NC). The resulting GdCo/NC exhibits a higher half‐wave potential of 0.861 V in alkaline media than that of Co/NC (0.824 V) and commercial Pt/C (0.843 V). The rechargeable Zn‐air battery assembled with GdCo/NC demonstrates great practical performance, delivering a high peak power density of 170.8 mW cm^−2^ and a large specific capacity of 733 mAh g^−1^, outperforming that of Pt/C + RuO_2_ benchmark. Theoretical calculations reveal that Gd incorporation modulates the local electronic structure of Co centers by inducing pronounced spin splitting and orbital‐selective spin polarization, which shifts the dominant spin polarization from d_z2_ orbital to d_yz_ orbital and lifts the degeneracy of d_xz_ and d_yz_ orbitals. Such spin‐dependent electronic reconstruction optimizes the adsorption of oxygen‐containing intermediates and shifts the rate‐determining step from *O protonation to *OH desorption, thus leading to excellent ORR performance.

## Graphical abstract

The Gd‐mediated spin‐state modulation strategy was proposed to enhance electrocatalytic performance of Co sites towards oxygen reduction reaction. The Gd incorporation modulates the local electronic structure of Co centers by inducing pronounced spin splitting and orbital‐selective spin polarization, which optimizes the adsorption of oxygen‐containing intermediates and shifts the rate‐determining step from *O protonation to *OH desorption, leading to excellent performance.

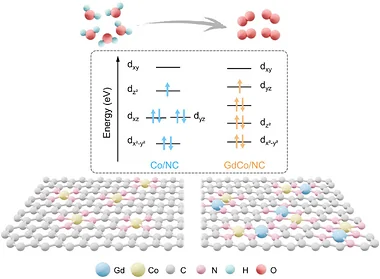

## 1 Introduction

The growing global demand for clean energy, together with the environmental burden associated with the extensive consumption of fossil fuels, has stimulated increasing interest in advanced energy‐storage systems and efficient energy‐conversion technologies [1, 2, 3]. Metal‐air batteries have attracted considerable attention owing to the high theoretical energy density, long operating life, and environmental compatibility [4, 5, 6]. The oxygen reduction reaction (ORR) generally proceeds through sluggish multistep proton/electron transfer processes accompanied by the formation and transformation of various oxygen‐containing intermediates, leading to slow reaction kinetics of metal‐air batteries [7, 8, 9]. Although Pt‐based materials remain the most representative ORR benchmarks, the scarcity and high cost of Pt, continue to impede their large‐scale deployment [10, 11].

In recent years, Co‐based materials have attracted considerable attention as promising electrocatalysts because of their low cost, abundant valence states, and tunable 3d electronic structures [12, 13]. The partially occupied 3d orbitals can hybridize with the antibonding orbitals of O_2_ molecules, facilitating interfacial electron transfer and weakening the O─O bond, thereby promoting O_2_ adsorption and activation [14, 15]. Such tunable d‐p orbital interaction endows Co‐based catalysts with considerable flexibility for regulating oxygen‐intermediate adsorption and optimizing ORR kinetics. Nevertheless, the intrinsic ORR activity of Co‐based catalysts suffers from the correlated adsorption energies of *OOH, *O, and *OH, which make it difficult to optimize the binding of these intermediates simultaneously [16, 17]. Spin polarization redistributes electrons among the metal d orbitals by altering the population of spin‐up and spin‐down states. This redistribution changes the occupation and energetic arrangement of the bonding and antibonding states, which in turn modifies the degree of hybridization between metal d orbitals and p orbitals of adsorbed intermediates [18, 19]. Consequently, inducing appropriate spin polarization can tune the strength of d‐p orbital hybridization and bring the adsorption energies of key intermediates closer to an energetically favorable range, thereby promoting the overall catalytic reaction. The incorporation of heteroelement therefore provides an effective strategy for inducing spin polarization and optimizing the adsorption energetics of key ORR intermediates [20, 21]. Zeng et al. introduced P into Fe─Co sites, inducing pronounced charge redistribution and modulating the 3d‐orbital energy levels of the metal centers [22]. In particular, P incorporation downshifted the Co 3d_z2_ orbital towards the O_2_ π* orbital, thereby improving Co─O orbital matching and stabilizing the bridge adsorption of O_2_. Beyond p‐block modulation, the incorporation of d‐block of transition metals has also attracted considerable attention, as the variable d‐electron configurations offer additional degrees of freedom for tailoring the orbital occupation of Co centers. For instance, the construction of Ir─Co atomic pairs induces pronounced redistribution of Co d electrons and alters the energy levels of specific 3d orbitals. The decreased occupation of Co d_z2_ orbital strengthens the Co─O interaction and optimizes the adsorption energetics of oxygen‐containing intermediates, thereby enhancing oxygen electrocatalytic activity [23, 24, 25].

Current studies on heterometallic regulation predominantly focused on electronic interactions arising from d‐d orbital coupling between transition‐metal centers [26, 27]. Rare‐earth (RE) elements possess unique 4f electronic configurations and strong polarization capabilities, making them promising electronic modifiers for regulating the local electronic and spin structures of neighboring Co centers [28, 29]. The unique 4f electronic configuration of RE elements enables f‐p‐d orbital coupling and interfacial charge redistribution with neighboring transition‐metal sites, which can alter ligand‐field splitting, d orbital occupation, and spin polarization, thereby providing an effective means for the adsorption and conversion of oxygen‐containing intermediates at catalytically active sites [30, 31, 32]. Recent studies demonstrated that coupling Tb with Co induces Co 3d‐O 2p‐Tb 4f orbital interactions, which reconstructs the electronic structure of Co centers and consequently optimizes the adsorption strengths of *OOH and *OH intermediates during ORR [33]. Similarly, Nd‐modified Co sites in N‐doped carbon could balance reversible oxygen electrocatalysis through Co 3d‐Nd 4f electronic coupling [34]. These results indicate that RE elements are able to regulate oxygen‐related adsorption processes through both electronic effect and oxophilic interaction. Previous studies mainly attributed the enhanced ORR activity of transition‐metal sites to RE‐induced f‐p‐d orbital coupling, whereas the influence of RE incorporation on the spin state of Co centers received far less attention.

Herein, we constructed a Gd‐modified Co─N─C catalyst (GdCo/NC) through a zeolitic imidazolate framework (ZIF)‐derived strategy to elucidate the regulatory role of Gd on the local electronic and spin structures of Co sites for alkaline ORR. The GdCo/NC exhibits excellent ORR activity with a high half‐wave potential of 0.861 V, which is higher than that of Co/NC (0.824 V) and commercial Pt/C (0.843 V). The GdCo/NC also presents good promising practical potential in rechargeable Zn‐air battery enabling a cell to achieve a peak power density of 170.8 mW cm^−2^ and a specific capacity of 733 mAh g^−1^. Theoretical calculations reveal that Gd incorporation induces spin splitting and redistributes the dominant spin polarization of Co from the d_z2_ to the d_yz_ orbital, thereby optimizing the adsorption of oxygen intermediates and shifting the rate‐determining step from *O protonation to *OH desorption. This work provides a good perspective on RE‐mediated electronic and spin regulation of Co─N─C catalysts for efficient oxygen electrocatalysis.

## 2 Results and Discussion

### 2.1 Synthesis and Characterization

GdCo/NC was synthesized through a zeolitic imidazolate framework‐8 (ZIF‐8)‐assisted impregnation‐pyrolysis strategy, as illustrated in Figure 1a. First, the GdCo‐ZIF precursor was obtained by dissolving Co(NO_3_)_2_·6H_2_O, Gd(NO_3_)_3_·6H_2_O, Zn(CH_3_COO)_2_, and 2‐methylimidazole (2‐MIM) in CH_3_OH under continuous stirring. The resulting GdCo‐ZIF precursor was then pyrolyzed at 900°C under an Ar atmosphere to produce GdCo/NC catalyst. For comparison, Co/NC was prepared using the same synthetic protocol in the absence of Gd(NO_3_)_3_·6H_2_O. Transmission and scanning electron microscopy (TEM and SEM) were employed to characterize the microstructure of catalysts. The pristine ZIF‐8 exhibits a well‐defined rhombic dodecahedral morphology (Figure S1). After the introduction of metal species, the morphology of GdCo‐ZIF and Co‐ZIF is well preserved without structure collapse (Figures S2 and S3). After pyrolysis, the GdCo/NC still retains the dodecahedral framework (Figure 1b,c). The high‐resolution TEM (HRTEM) image of GdCo/NC reveals a typical amorphous carbon structure without observable lattice fringes (Figure 1d). The selected area electron diffraction (SAED) pattern displays a diffuse halo feature without diffraction spots, which excludes the formation of crystalline Gd‐ or Co‐containing aggregates. The aberration‐corrected high‐angle annular dark‐field scanning TEM (AC‐HAADF‐STEM) image shows that numerous isolated bright spots are uniformly distributed over the carbon matrix (Figure 1e), indicating the atomic‐level dispersion of Gd and Co species in GdCo/NC. Nitrogen adsorption‐desorption isotherm measurements confirm the porous carbon framework for GdCo/NC and Co/NC [35], which exhibit high Brunauer–Emmett–Teller (BET) specific surface areas of 2095.96 and 1613.94 m^2^ g^−1^ respectively (Figure S4). Such textural characteristics can expose more accessible surface sites and facilitate electrolyte penetration and mass transport during electrocatalysis [36]. To investigate the spatial distribution of the constituent elements in GdCo/NC, HAADF‐STEM image and corresponding energy‐dispersive spectroscopy (EDS) elemental mappings were performed. As shown in Figure 1f,g, Gd, Co, N, and O signals are homogeneously distributed throughout the carbon framework. Inductively coupled plasma mass spectrometry (ICP‐MS) analysis determines the Gd and Co contents in GdCo/NC to be 1.40 and 2.58 wt.% respectively. The Co/NC exhibits the homogeneous incorporation of Co species within the N‐doped carbon framework (Figure S5).

**FIGURE 1:**
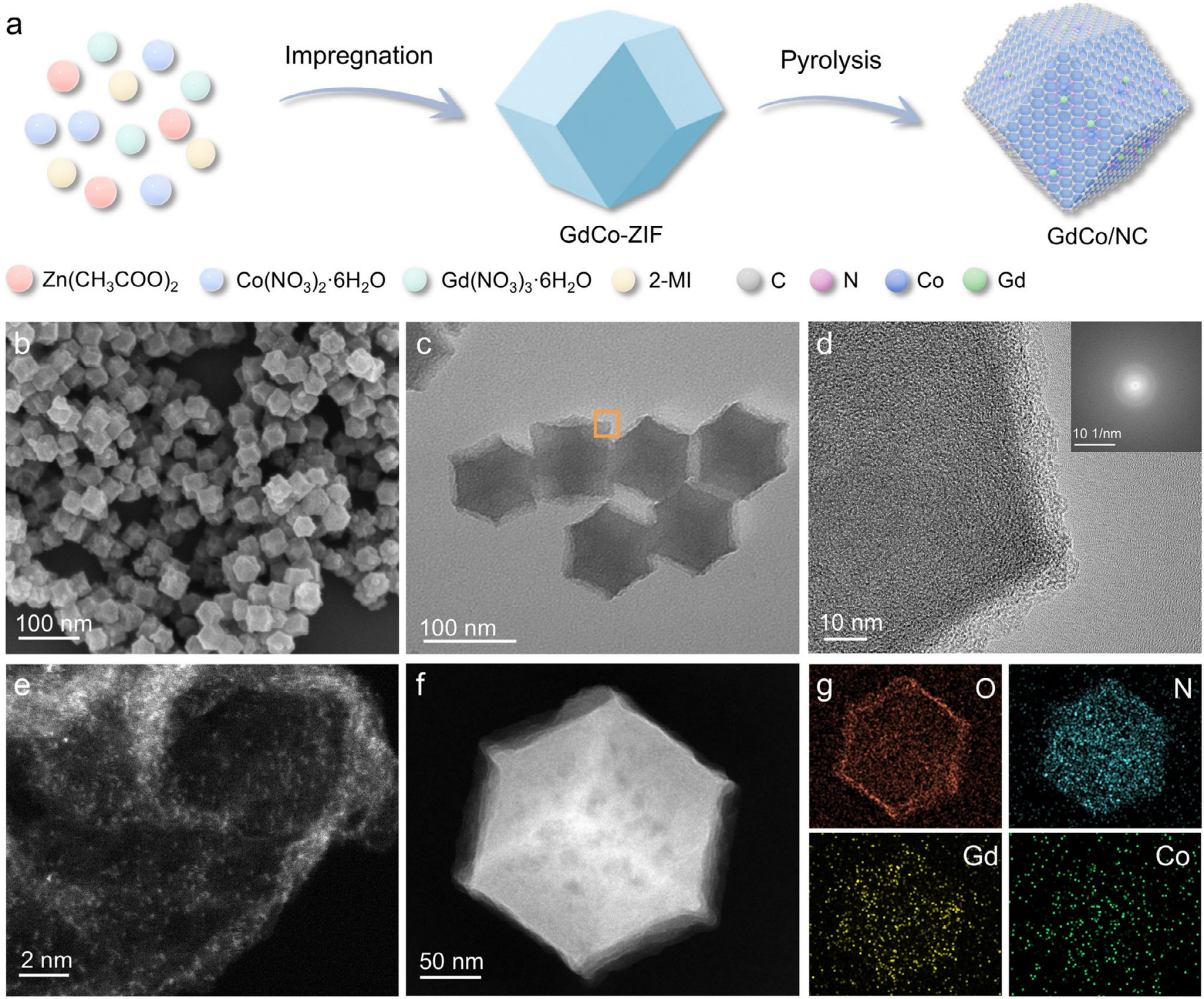
(a) Schematic illustration of the preparation for GdCo/NC; (b) SEM image of GdCo/NC; (c) TEM and (d) HRTEM image of GdCo/NC, the inset in pattern (d) is SAED pattern; (e) AC HAADF‐STEM image of GdCo/NC; (f) HAADF‐STEM image of GdCo/NC and (g) the corresponding EDX element mappings.

X‐ray diffraction (XRD) patterns of GdCo/NC and Co/NC show two broad diffraction peaks centered at about 26.5° and 42.8° (Figure 2a), which are indexed to the (002) and (100) planes of graphitic carbon respectively [37]. The absence of diffraction peaks associated with Gd‐ or Co‐containing phases confirms the highly dispersed Gd and Co species within the NC framework. Raman spectroscopy was employed to evaluate the degree of disorder and graphitization of NC framework. As presented in Figure 2b, both GdCo/NC and Co/NC exhibit two characteristic bands at around 1350 and 1580 cm^−1^, corresponding to D‐band associated with defective/disordered carbon and G‐band originating from graphitic sp^2^‐hybridized carbon, respectively [38, 39]. The GdCo/NC shows the lower I_D_/I_G_ value (1.01) than that of Co/NC (1.24), indicating a higher degree of graphitization in GdCo/NC. The incorporation of Gd may regulate the carbonization process and promote the formation of more ordered sp^2^‐hybridized carbon domains [40]. The improved graphitic ordering is expected to enhance the electrical conductivity of catalysts, facilitating interfacial charge transfer and ORR kinetics [41]. The surface chemical composition and elemental state of catalysts were investigated via X‐ray photoelectron spectroscopy (XPS). As shown in Figure S6, the survey scan XPS spectrum confirms the co‐existence of Gd, Co, N, and C elements. For high‐resolution Gd 4d spectrum (Figure 2c), two pronounced peaks at 140.5 and 152.1 eV are assigned to Gd 4d_5/2_ and Gd 4d_3/2_ orbitals, respectively [42]. An extra peak observed at 145.7 eV is attributed to the multiplet‐splitting component associated with the Gd 4d level. For high‐resolution Co 2p spectrum (Figure 2d), the main peaks for 2p_3/2_ and 2p_1/2_ are located at 780.3 and 795.8 eV respectively, which can be resolved into Co^3+^ and Co^2+^ species, respectively [43, 44]. Compared with Co/NC, the Co 2p_3/2_ peak of GdCo/NC shifts positively by about 0.23 eV towards higher binding energy. The fitted Co 2p spectra reveal an increased Co^2+^/Co^3+^ ratio in GdCo/NC, suggesting that the Gd incorporation redistributes the Co valence states towards a higher proportion of Co^2+^ species. This result suggests the interaction between Gd species and Co sites modulates the local electronic structure of Co centers [15]. As shown in Figure 2e, the N 1s spectra of both catalysts are deconvoluted into pyridinic N, Co─N, pyrrolic N, and graphitic N species [45], and the corresponding relative contents are summarized in Figure S7. Compared with Co/NC, the Co─N peak in GdCo/NC shifts positively by about 0.5 eV towards higher binding energy, indicating the changed electronic distribution of Co─N coordination environment after the Gd incorporation [42]. The Gd‐induced redistribution of Co valence states is closely associated with the spin state. The Co^2+^ with a 3d^7^ electronic configuration can favor a high‐spin state under an appropriate ligand field, giving rise to a larger population of unpaired electrons [46]. Figure 2f shows the magnetization‐hysteresis (*M‐H*) curves of Co/NC and GdCo/NC measured at ambient temperature. The GdCo/NC shows a stronger magnetization response than that of Co/NC, indicating that the Gd incorporation increases the number of unpaired electrons in Co/NC, which benefits to the spin‐selected ORR process [47].

**FIGURE 2:**
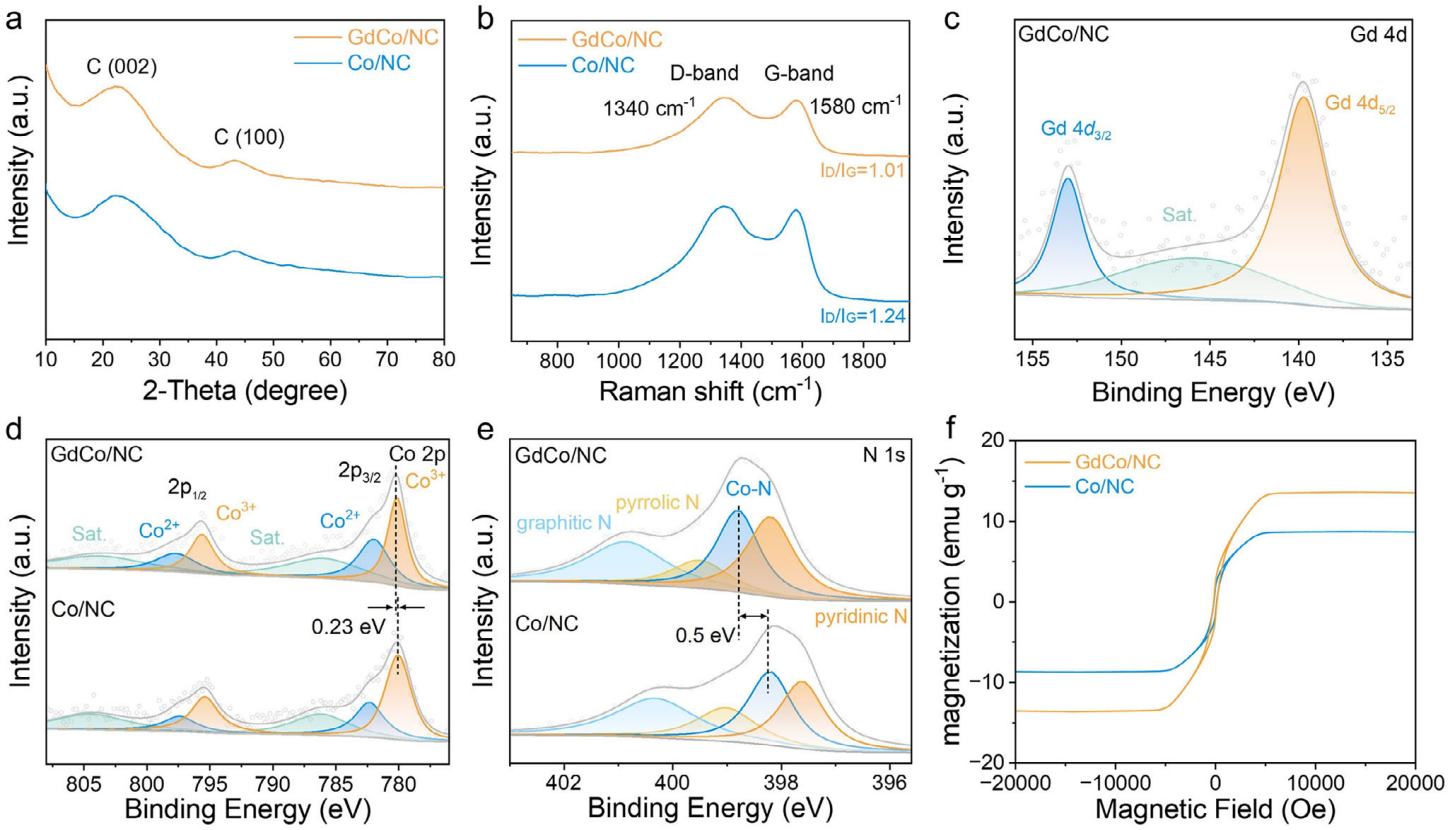
(a) XRD patterns of GdCo/NC and Co/NC; (b) Raman spectra of GdCo/NC and Co/NC; high‐resolution (c) Gd 4d, (d) Co 2p, (e) N 1s XPS spectra of GdCo/NC and Co/NC; (f) Magnetic‐field‐dependent magnetization at ambient temperature of GdCo/NC and Co/NC.

### 2.2 Electrocatalytic Oxygen Reduction Performance

The electrochemical measurements were carried out in O_2_‐saturated 0.1 M KOH solution using a rotating ring‐disk electrode (RRDE). All potentials were calibrated to the reversible hydrogen electrode (RHE) scale and corrected with iR compensation. To obtain the optimal GdCo/NC, the pyrolysis temperature was screened (Figures S8 and S9). The catalyst prepared at 900°C exhibits superior ORR activity compared with those prepared at 800°C and 1000°C (Figure S10). The GdCo/NC samples with varied Gd loadings were also synthesized to explore the influence of Gd content on ORR activity. (Figures S11 and S12). The GdCo/NC with a Gd/Co ratio of 1/5 delivers the highest half‐wave potential (*E*_1/2_) of 0.861 V among GdCo/NC samples (Figure 3a), outperforming those with Gd/Co ratios of 1/1 and 1/10. The ORR activity of the optimal GdCo/NC was then compared with that of Co/NC, Gd/NC and commercial Pt/C (Figure 3b). The Gd/NC exhibits much lower ORR activity than GdCo/NC, indicating that Gd alone does not serve as the dominant active site towards ORR (Figure S13). The GdCo/NC displays excellent ORR polarization behavior with *E*_1/2_ of 0.861 V and the kinetic current density (*J*_k_) of 7.39 mA cm^−2^ at 0.85 V (Figure 3c), which are higher than those of commercial Pt/C (*E*_1/2_ = 0.843 V, *J*_k_ = 4.79 mA cm^−2^) and Co/NC (*E*_1/2_ = 0.824 V, *J*_k_ = 2.52 mA cm^−2^), suggesting that the Gd incorporation enhances ORR activity. Further kinetic analysis shows that the GdCo/NC exhibits a Tafel slope of 89.45 mV dec^−1^ (Figure 3d), which is lower than those of Pt/C (101.12 mV dec^−1^) and Co/NC (113.05 mV dec^−1^), indicating that the Gd incorporation enhances the ORR kinetics. Furthermore, the GdCo/NC exhibits superior ORR activity compared with recently reported electrocatalysts (Table S1). Electrochemical impedance spectroscopy (EIS) was employed to investigate the interfacial charge‐transfer behavior of catalysts in ORR. As shown in Figure 3e, the GdCo/NC exhibits a smaller charge transfer resistance (*R*_ct_) than Co/NC, indicating that the Gd incorporation is beneficial for improving charge transfer at the catalyst/electrolyte interface. The electrochemically active surface area (ECSA) was estimated from the double‐layer capacitance (*C*_dl_) measured in the non‐Faradaic potential region (Figure 3f and Figure S14). The *C*_dl_ value of GdCo/NC is 25.80 mF cm^−2^, which is 1.35 times that of Co/NC (19.16 mF cm^−2^), suggesting a larger electrochemically accessible surface area for GdCo/NC. The kinetic current density was normalized by ECSA and BET surface to exclude the influence of the electrochemically accessible surface areas. The GdCo/NC exhibits the higher ECSA‐ and BET‐normalized kinetic current densities than those of Co/NC over the potential range (Figure S15), indicating that the accelerated ORR kinetics originate from the electronically modified Co centers. In addition to catalytic activity, the ORR selectivity is another important criterion for evaluating electrocatalysts. RRDE measurements reveal that the GdCo/NC delivers an electron‐transfer number (n) of about 3.97 over the tested potential range, accompanied by an H_2_O_2_ yield below 5% (Figure 3g), indicating a predominant four‐electron oxygen reduction pathway, in accordance with the Koutecky‐Levich result (Figures S16 and S17). The ORR stability of GdCo/NC was evaluated by accelerated durability tests (ADT) and chronoamperometric *i‐t* measurements. The GdCo/NC shows negligible decay of half‐wave potential after 5000 ADT cycles (Figure 3h), demonstrating good cycling stability. After continuous *i‐t* measurement for 12 h, the GdCo/NC exhibits only a 3.77% decrease in current density (Figure 3i), which is much lower than those of Pt/C (25.53%) and Co/NC (21.59%). After the *i‐t* test, the structure and composition of GdCo/NC are well preserved compared with that of the initial sample, whereas the Co/NC shows slight structure degradation (Figures S18 and S19). The chemical states of Gd and Co in the recovered GdCo/NC exhibit no obvious changes relative to the initial catalysts, demonstrating that these chemical states remain stable during ORR operation (Figures S20 and S21). ICP analysis reveals that the Gd and Co contents after the *i‐t* test remain at 1.24 and 2.21 wt.% respectively, confirming the negligible dissolution of metal species.

**FIGURE 3:**
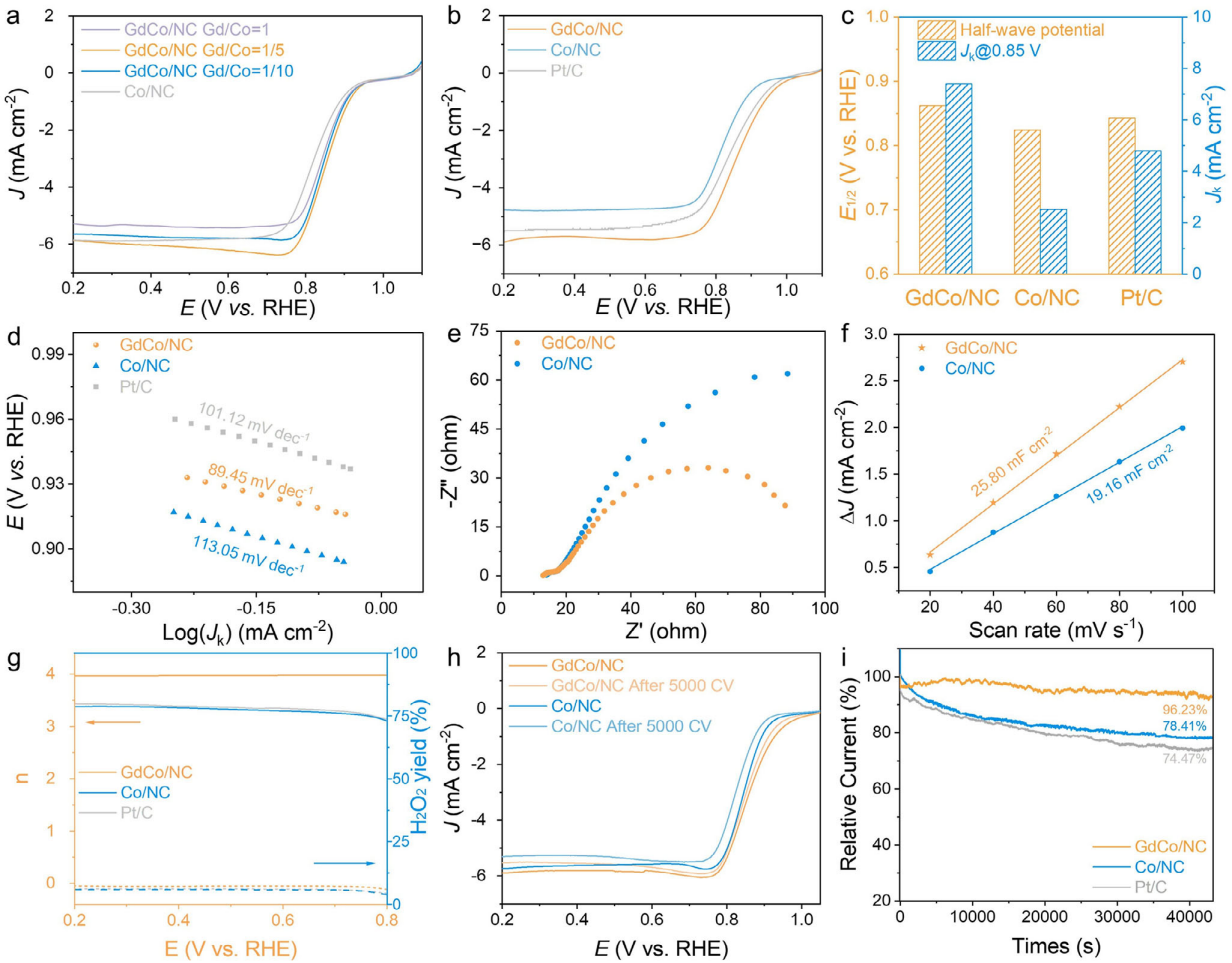
(a) LSV curves of GdCo/NC with different Gd contents; (b) LSV curves of GdCo/NC, Co/NC and Pt/C; (c) Comparison of *E*_1/2_ and *J*_k_ at 0.85 V for catalysts; (d) Tafel plots of GdCo/NC, Co/NC and Pt/C; (e) Nyquist plots of GdCo/NC and Co/NC; (f) Electrochemical double‐layer capacitance of GdCo/NC and Co/NC; (g) H_2_O_2_ production yields and electrons transfer number (n) of GdCo/NC, Co/NC and Pt/C; (h) The ORR polarization curves after 5000 cycles for GdCo/NC and Co/NC; (i) The *i‐t* curve of GdCo/NC, Co/NC and Pt/C.

### 2.3 Theoretical Insights and DFT Calculations

Density functional theory (DFT) calculations were performed to elucidate the regulation mechanism of the Gd introduction on the microenvironment of Co/NC active center. Based on previous reports [48, 49], three representative Gd‐Co configurations with different relative positions and local coordination environments were constructed. The formation energy (*E*_form_) of various candidate configurations after full structural relaxation were calculated to evaluate the thermodynamic stability (Figure S22). Among these configurations, Figure S22a exhibits the lowest formation energy (*E*_form_ = −8.92 eV), indicating that it is the most thermodynamically favorable. Therefore, this structure was selected as the representative model for the subsequent DFT calculations and mechanistic analyses. As shown in Figure 4a, in the GdCo/NC model, Gd is positioned adjacent to the Co center, creating a chemically non‐equivalent local environment and enabling electronic interaction between two metal species. The electron localization function (ELF) was analyzed to probe the local electronic characteristics of Co/NC and GdCo/NC. As shown in Figure S23, the introduction of Gd leads to a pronounced redistribution of the localized electron density in the vicinity of the Co center, indicating a substantial perturbation of the local electronic environment. Meanwhile, strong electron localization was observed around the Gd site, which is consistent with the highly localized nature of Gd 4f electrons. The charge density difference analysis reveals obvious electron accumulation around the Co center in GdCo/NC, indicating electron donation from Gd to the adjacent Co site (Figure 4b). The charge redistribution was further quantified by Bader charge analysis. The Bader charge of the Co site changes from +1.00 |e| in Co/NC to +0.98 |e| in GdCo/NC (Figure S24), indicating a slight increase in the electron density around the Co center, which is consistent with the XPS results. To further investigate the influence of Gd‐induced local electronic modulation on the catalytic process, the Gibbs free‐energy profiles of ORR pathways on GdCo/NC and Co/NC were calculated (Figure 4c). The optimized adsorption configurations of the other oxygen‐containing intermediates are presented in Figures S25 and S26. At the bias potential of U = 0 V, all elementary ORR steps on both GdCo/NC and Co/NC are thermodynamically spontaneous. When the bias potential increases to 1.23 V, the rate‐determining step (RDS) for Co/NC is the protonation of *O to generate an adsorbed *OH with a high energy barrier of 0.60 eV. In contrast, the RDS for GdCo/NC shifts to *OH desorption, with a low free energy barrier of 0.40 eV, indicating that the Gd incorporation effectively optimizes the adsorption strength of ORR intermediates and improves the intrinsic ORR thermodynamics. To further verify the electronic modulation effect of Gd on the Co active center, projected density of states (PDOS) calculations was performed. As shown in Figure 4d, the Gd 4f states are mainly located in the deep energy level, reflecting the highly localized electronic characteristics of Gd. For Co/NC, no obvious occupation of Co 3d states was observed near the Fermi level, whereas GdCo/NC exhibits pronounced Co 3d occupation around the Fermi level. This result indicates that the Gd incorporation effectively modulates the electronic structure of Co and increases the density of reactive electronic states near the Fermi level, which is beneficial for charge transfer and orbital interaction with oxygen‐containing ORR intermediates. In addition, the spin‐resolved PDOS reveals the spin splitting of Co 3d states after the Gd incorporation. The spin‐up and spin‐down states of Co are located at −2.57 and −1.10 eV for Co/NC, while they shift to −2.80 and −0.70 eV for GdCo/NC (Figure 4e), respectively, which demonstrates the enhanced spin asymmetry of Co induced by Gd. To gain fundamental insight into the origin of the enhanced ORR activity of GdCo/NC, the electronic filling and energy level arrangement of Co 3d orbitals were systematically investigated. Figure 4f,g show the calculated PDOS of different d‐electron orbitals for Co/NC and GdCo/NC. The spin polarization for Co/NC is mainly dominated by the d_z2_ orbital, whereas in GdCo/NC, the d_z2_ orbital becomes more occupied and nearly spin‐paired, leading to a markedly reduced spin‐polarization contribution. Meanwhile, in Figure 4h, the d_xz_ and d_yz_ orbitals in GdCo/NC exhibit a lifting of degeneracy, accompanied by an upward shift in the energy levels. The upward shift and degeneracy lifting of the d_xz_/d_yz_ orbitals, together with enhanced orbital‐selective spin polarization, may increase the ability of the Co center to electronically couple with oxygenated ORR intermediates. The modulation of the spin characteristics is further supported by the spin‐density distributions (Figure S27). After the introduction of Gd, the spin density at the Co site evolves into a pronounced d_yz_ distribution from a relatively symmetric d_z2_ feature.

**FIGURE 4:**
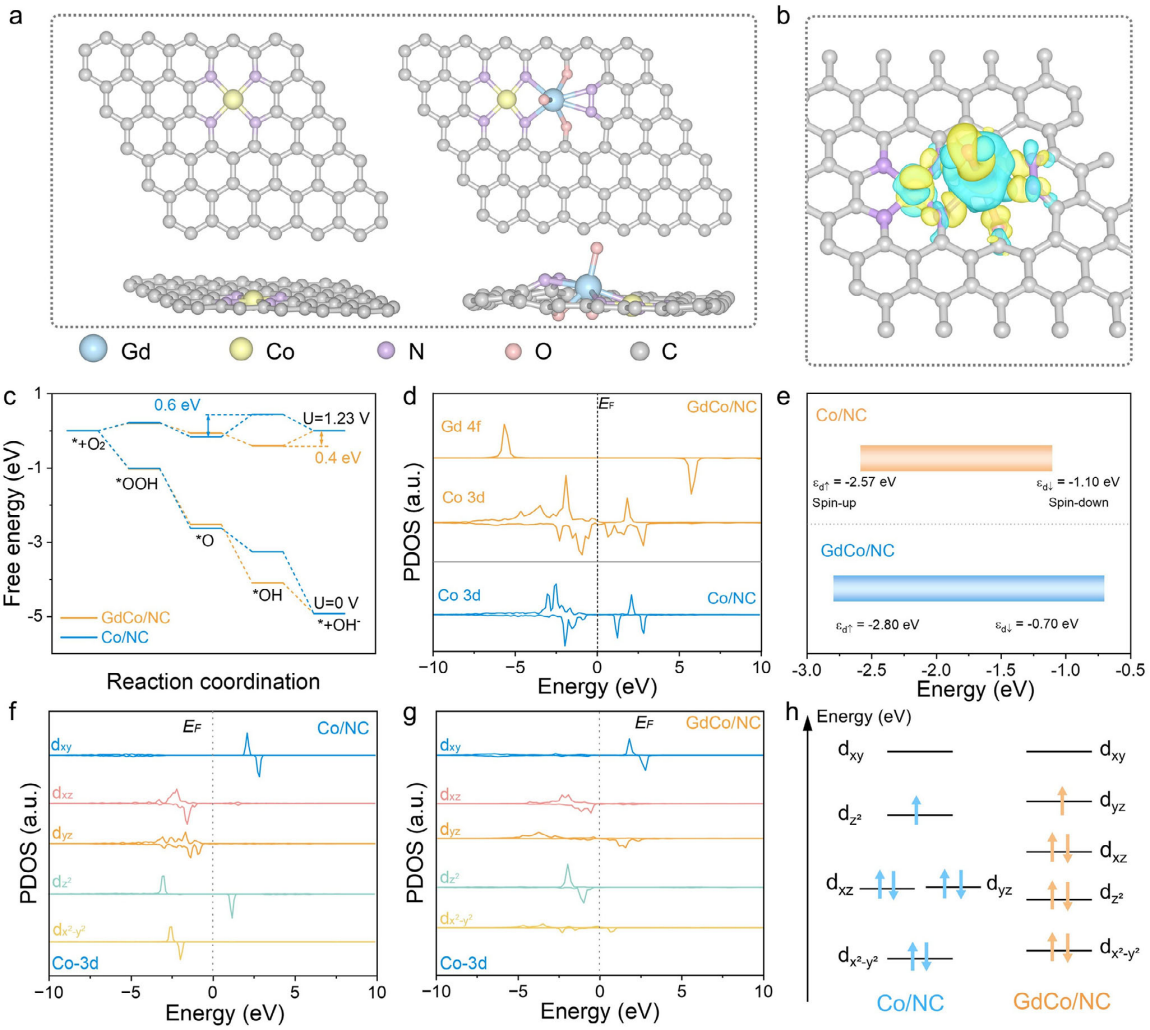
(a) Schematic model of Co/NC and GdCo/NC; (b) Charge density difference analysis of GdCo/NC; (c) Free energy pathway of ORR for GdCo/NC and Co/NC; (d) PDOS for Gd 4f and Co 3d of GdCo/NC and Co/NC; (e) Schematic comparison of d‐band centers for Co/NC and GdCo/NC; (f) The corresponding orbital projected states of Co 3d for (f) Co/NC and (g) GdCo/NC; (h) Co 3d orbital splitting of Co centers in Co/NC and GdCo/NC.

### 2.4 Zn‐Air Battery Performance

Apart from outstanding ORR performance, the GdCo/NC also exhibits good electrocatalytic activity and stability towards oxygen evolution reaction (OER) in 0.1 M KOH electrolyte, as verified by OER measurements (Figures S28 and S29) and the corresponding DFT calculations (Figure S30). In light of the bifunctional activity of GdCo/NC, the as‐prepared catalyst was further employed as an air‐cathode catalyst in rechargeable Zn‐air batteries to evaluate its practical application potential. The assembled Zn‐air battery used a polished Zn plate as the anode, a gas‐diffusion electrode loaded with GdCo/NC as the air cathode, and a mixed electrolyte containing 6 M KOH and 0.2 M ZnCl_2_ (Figure 5a) [50, 51]. The Pt/C + RuO_2_‐based cell as the reference was also tested. As shown in Figure 5b, the GdCo/NC‐based cell exhibits a stable open‐circuit voltage of 1.493 V, higher than that of Pt/C + RuO_2_‐based cell (1.401 V). Galvanostatic charge‐discharge measurements reveal that the GdCo/NC‐based cell shows a smaller charge‐discharge voltage gap than Pt/C + RuO_2_‐based cell, suggesting lower electrochemical polarization during battery operation (Figure 5c). In the discharge polarization and power‐density curves, the GdCo/NC‐based cell achieves a peak power density of 170.8 mW cm^−2^ at 209.9 mA cm^−2^ (Figure 5d), which is about 1.7 times that of Pt/C + RuO_2_‐based cell (102.5 mW cm^−2^ at 154.53 mA cm^−2^). These results demonstrate that the GdCo/NC‐based air cathode enables superior discharge output capability. To further examine the discharge adaptability under different load conditions, the discharge voltage profiles were recorded at various current densities. As shown in Figure 5e, the discharge voltages of both Zn‐air cells decrease with increasing current density and then gradually stabilize at the corresponding current density. In comparison, the GdCo/NC‐based cell maintains higher discharge voltages than Pt/C + RuO_2_‐based cell at all tested current densities, indicating better rate discharge performance. Moreover, the specific capacity of GdCo/NC‐based cell reaches 733 mAh g^−1^ (Figure 5f), markedly higher than that of Pt/C + RuO_2_‐based cell (547 mAh g^−1^), further confirming its enhanced sustained discharge capability. Finally, the cycling stability of the GdCo/NC‐based and Pt/C + RuO_2_‐based cells were evaluated at a charge‐discharge current density of 5 mA cm^−2^. As shown in Figure 5g, the GdCo/NC‐based cell maintains stable alternating charge‐discharge operation over 800 h, outperforming that of Pt/C + RuO_2_‐based cell. The enlarged charge‐discharge profiles show that the initial voltage gap of the GdCo/NC‐based cell is only 0.79 V within the first 5 h, corresponding to a round‐trip efficiency of 60.2% (Figure S31). After the operation of 325 h, the voltage gap increases only slightly to 0.85 V, while the round‐trip efficiency remains at 57.2%. Even after prolonged operation for 775 h, the device still maintains reversible charge–discharge behavior with a voltage gap of 1.24 V and a round‐trip efficiency of 40.6%. These results demonstrate good operational stability of GdCo/NC‐based cell during long‐term cycling, highlighting its promising potential for practical rechargeable cell applications.

**FIGURE 5:**
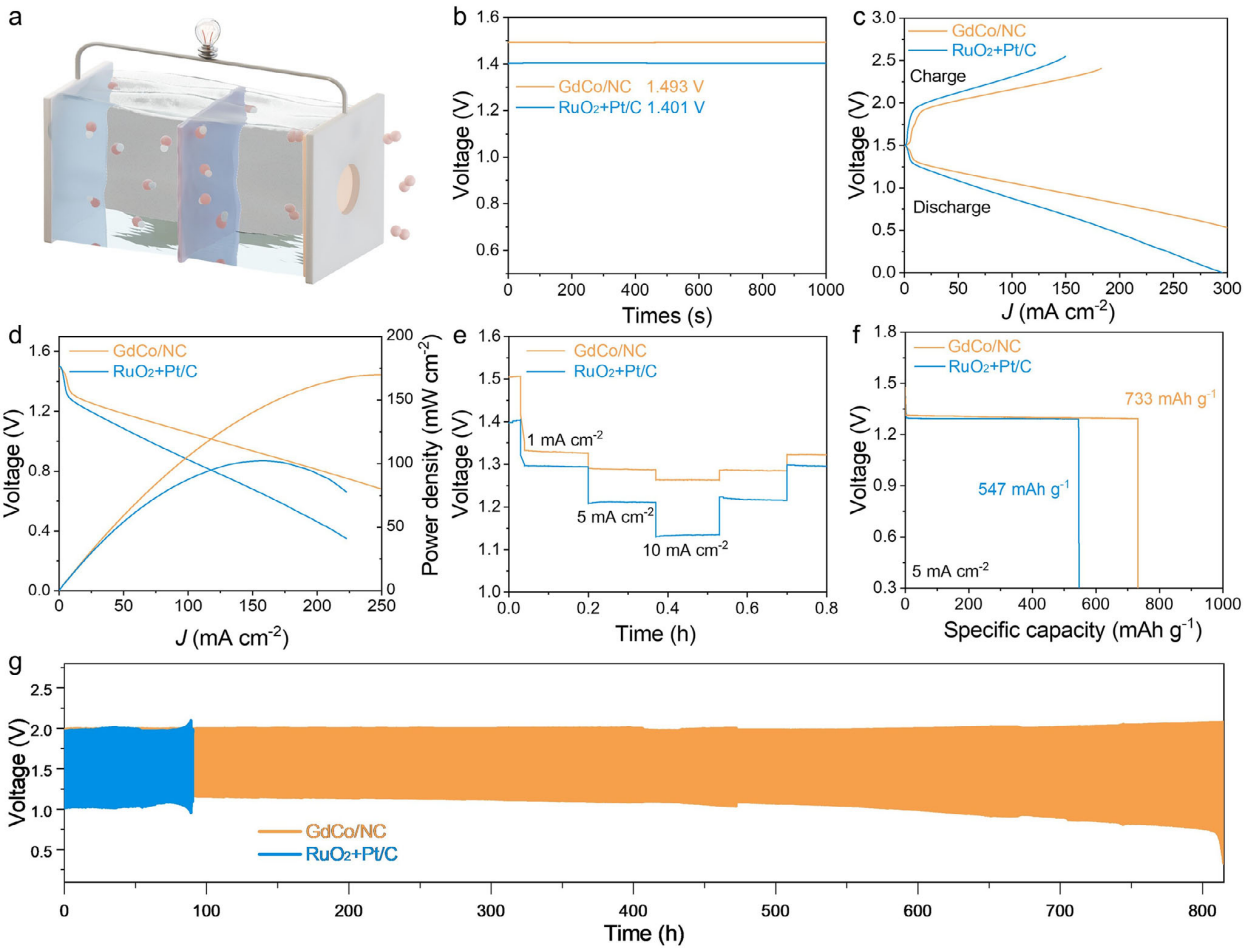
(a) Schematic diagram of home‐made Zn‐air battery model; (b) Open‐circuit potential curves for GdCo/NC and Pt/C + RuO_2_‐based cells; (c) Polarized curves of charge and discharge processes; (d) Power density curves for discharge; (e) Discharge process at different current densities; (f) Specific capacity of GdCo/NC and Pt/C + RuO_2_‐based cells; (g) Galvanostatic cycling of the rechargeable GdCo/NC and Pt/C + RuO_2_‐based cells at 5 mA cm^−2^.

## 3 Conclusion

In summary, a GdCo/NC catalyst was constructed through a ZIF‐derived strategy to investigate the regulatory role of Gd in modulating the local electronic and spin structures of Co sites during alkaline ORR. The GdCo/NC exhibits excellent alkaline ORR activity, with a half‐wave potential of 0.861 V, outperforming commercial Pt/C and Co/NC. Besides, the GdCo/NC‐based rechargeable Zn‐air battery delivered a high peak power density of 170.8 mW cm^−2^ and a large specific capacity of 733 mAh g^−1^. Theoretical calculations revealed that Gd incorporation modulated the local electronic structure of Co centers, inducing pronounced spin splitting and orbital‐selective spin polarization. The dominant spin polarization is redistributed from the d_z2_ orbital to the d_yz_ orbital, while the degeneracy between the d_xz_ and d_yz_ orbitals was lifted. The electronic changes optimized the adsorption of oxygen‐containing intermediates and shifted the rate‐determining step from *O protonation to *OH desorption. This work provides a new perspective on RE‐mediated electronic and spin‐state regulation of Co centers and offers a rational strategy for designing high‐performance oxygen electrocatalysts.

## Author Contributions

**Yijie Shen**: investigation, writing – original draft. **Yu Zhu**: investigation, writing – original draft. **Tianrui Zhao**: methodology, software. **Yiwen An**: conceptualization, data curation. **Xuan Wang**: software, formal analysis. **Meng Li**: validation, visualization. **Dongmei Sun**: supervision. **Linda Zhang**: writing – review and editing. **Hao Li**: writing – review and editing, software, funding acquisition. **Yawen Tang**: supervision, project administration. **Gengtao Fu**: supervision, project administration, funding acquisition, writing – review and editing.

## Conflicts of Interest

The authors declare no conflicts of interest.

## Supporting information




**Supporting File**: advs78659‐sup‐0001‐SuppMat.docx.

## Data Availability

The data that support the findings of this study are available from the corresponding author upon reasonable request.
