# Supplementary material for: Gd‐Mediated Spin‐State Modulation of Co Sites Enhances Oxygen Electrocatalysis in Zn‐Air Battery
Source: Adv Sci (Weinh). 2026 Nov 5:e78659. Online ahead of print. doi: 10.1002/advs.78659 (PMC13735430; doi:10.1002/advs.78659)
Supplement: Supplementary file 1 [file ADVS-9999-e78659-s001.docx]

**Supporting information**

**Gd-Mediated Spin-State Modulation of Co Sites Enhances Oxygen Electrocatalysis** **in Zn-Air Battery**

Yijie Shen^1,#^, Yu Zhu^1,#^, Tianrui Zhao^1^, Yiwen An^1^, Xuan Wang^1^, Meng Li^1^, Dongmei Sun^1^, Linda Zhang^2^, Hao Li^2,^*, Yawen Tang^1,^*, Gengtao Fu^1,^*

^1^ Jiangsu Key Laboratory of Long-duration Energy Storage Battery Technology, Jiangsu Key Laboratory of Micro Nano Sensing and Separation Science for Analytical Chemistry, School of Chemistry and Materials Science, Nanjing Normal University, Nanjing 210023, China.

^2^ Advanced Institute for Materials Research (WPI-AIMR), Tohoku University, Sendai 980-8577, Japan.

*Email: li.hao.b8@tohoku.ac.jp (H. Li); tangyawen@njnu.edu.cn (Y. Tang); gengtaofu@njnu.edu.cn (G. Fu)

**Experimental Section**

**Chemicals and reagents**

The gadolinium (III) nitrate hexahydrate (Gd(NO_3_)_3_·6H_2_O), 2-methylimidazole (2-MIM), Cobalt nitrate hexahydrate (Co(NO_3_)_2_·6H_2_O), Zinc acetate (Zn(CH_3_COO)_2_) were obtained from Aladdin Ltd. (Shanghai, China). Methanol (MeOH) was obtained from Sinopharm Chemical Reagent Co., Ltd. (Shanghai, China). Commercial RuO_2_ and 20% Pt/C were purchased from Johnson Matthey Corporation. All the reagents were of analytical reagent grade and used without further purification.

**Synthesis of GdCo-ZIF**

In a typical synthesis, Zn(CH_3_COO)_2_ (0.2615 g), Co(NO_3_)_2_·6H_2_O (0.055 g), and Gd(NO_3_)_3_·6H_2_O (16.94 mg) were dissolved in 15 mL of MeOH to form solution A. Meanwhile, 0.616 g of 2-MI was dissolved in another 15 mL of MeOH to obtain solution B. The two solutions were then mixed thoroughly and stir at room temperature for 24 h. Subsequently, the GdCo-ZIF product was collected by high-speed centrifugation, washed several times with MeOH, and dried under vacuum at 50 ^o^C overnight. For comparative purposes, Co-ZIF was synthesized using the same procedure, except Gd(NO_3_)_3_·6H_2_O was excluded from the synthesis.

**Synthesis of GdCo/NC**

The obtained GdCo-ZIF precursor was placed in a porcelain crucible and thermally treated at 900 ^o^C for 180 min under an Ar atmosphere with a controlled heating rate of 5 ^o^C min^-1^, yielding the final GdCo/NC product. The Co/NC sample was prepared using the same thermal treatment procedure.

**Physical characterizations**

X-ray powder diffraction (XRD) patterns were carried on a D/max-rC X-ray diffractometer with Cu Kα radiation source (λ = 1.5406 Å) and manipulated at 40 kV and 100 mA. Transmission electron microscopy (TEM) and scanning electron microscope (SEM) images were captured by JEOL JEM-2100F and JEOL JSM7500F, respectively. High-resolution transmission electron microscopy (HRTEM), high-angle annular dark-field scanning transmission electron microscopy (HAADF-STEM), and energy dispersive X-ray spectrum (EDX) were acquired on JEOL JEM-2100F with an accelerating voltage of 200 kV. The Brunauer-Emmett-Teller (BET) specific surface area and pore size distribution were carried out under 77 K with a Micromeritics ASAP 2020 system.

**Electrochemical Measurements**

All electrochemical measurements were conducted using a CHI 760E electrochemical analyzer (Chenhua, Shanghai) with a typical three-electrode system. To facilitate comparison and analysis, all measured potentials were converted to the reversible hydrogen electrode (RHE) scale using the equation *E*_RHE_ = *E*_SCE_ + 0.05916*pH + 0.242. The electrocatalyst ink was prepared by mixing 5 mg of electrocatalyst with 1 mL of solution. The solution was composed of deionized water, ethanol, and Nafion in a volume ratio of 7: 2: 1. The resulting mixture was sonicated for approximately 30 min to ensure homogeneity. For electrochemical measurements, a carbon electrode was used as the auxiliary electrode, a saturated calomel electrode (SCE) served as the reference electrode, and a rotating ring disc electrode with a surface area of 0.196 cm^2^ was employed as the working electrode. To load the ink onto the rotating ring disc electrode for oxygen reduction reaction (ORR) testing, 10 μL of the ink was applied. Linear scanning voltammetric (LSV) curves were recorded in O_2_-saturated 0.1 M KOH electrolyte at a scan rate of 5 mV s^-1^ and a temperature of 25 ^o^C. And the *J*_K_ is kinetic current density. The chronoamperometric *i-t* curves were obtained at 0.75 V vs. RHE for catalysts in O_2_-saturated 0.1 M KOH electrolyte under 1600 rpm for 12 h. The electrochemical impedance spectroscopy (EIS) was recorded in O_2_-saturated 0.1 M KOH at the open voltage from 10 kHz to 0.1 Hz without rotating speed. The selectivity of the electrocatalyst during the ORR process was determined by measuring LSV curves on RDE with different rotating speeds (400, 625, 900, 1225, 1600 and 2025 rpm). The percentage of hydrogen peroxide yield (%HO_2_^-^) and the value of n were calculated using equations (1) and (2), respectively.

${\%HO}_{2}^{-}=200*\frac{\frac{i_{r}}{N}}{i_{d}+\frac{i_{r}}{N}}$	(1)

$n=4*\frac{\frac{i_{r}}{N}}{i_{d}+\frac{i_{r}}{N}}$	(2)

where *i_r_* and *i_d_* are the ring and disk current obtained from the RRDE test, respectively. The N is the ring current collection efficiency (*N* = 0.37).

**Theoretical calculations**

All spin-polarized density functional theory (DFT) calculations were performed using the Vienna *ab initio* Simulation Package (VASP).^[1, 2]^ The ion-electron interactions were described by the projector augmented-wave (PAW) method.^[3, 4]^ The exchange–correlation effects were treated within the generalized gradient approximation (GGA) using the revised Perdew-Burke-Ernzerhof (RPBE) functional.^[5-8]^ Wavefunction for valence electrons were expanded by Kohn-Sham plane-wave basis set with the kinetic energy cutoff of 400 eV.^[9-11]^ The convergence criterion for geometric relaxation assumes that the Hellmann-Feynman forces on each atom should be lower than0.03 eV/Å. For energy minimization, the convergence criterion for wavefunction optimization accepts the value of 10^-5^. A *k*-point mesh of 2×2×1 with the Gamma-centered sampling in the Brillouin zone was used for the slab model, which is exerted with a vacuum layer of 15 Å to avoid the periodic interaction along the c direction. The bulk structure was loaded from the Materials Project database.^[12, 13]^ To deal with the strongly on-site coulomb interaction associated with localized 3d and 4f electrons, the Hubbard U correction by Dudarev et al.^[7]^ was introduced by considering the effective U value (U_eff_), where 2.0 eV and 6.0 eV are adopted for Co 3d and Gd 4f respectively according to previous works.^[14, 15]^ The free energy was calculated according to the computational hydrogen electrode (CHE) by Nørskov et al.^[16]^ Due to the DFT error for the energy of triplet-state of O_2_, the experimental value of 4.92 eV under standard condition for the whole ORR process was used to retract the self-consistent free energy of O_2_. Zero-point energy and entropy corrections were taken from previous studies.

**Zn-air Battery Test**

The liquid zinc-air battery tests were conducted using a homemade zinc-air battery setup. The electrocatalyst ink was prepared by mixing 10 mg of electrocatalyst with 250 μL of solution. The solution was prepared by combining 100 μL of deionized water, 100 μL of methanol, and 50 μL of Nafion. This mixture ensures the proper dispersion of the electrocatalyst. The air cathode of the battery was composed of a porous carbon paper and a gas diffusion layer (GDL). The alkaline electrolyte utilized for the rechargeable zinc-air cell consisted of 0.2 M ZnCl_2_ and 6 M KOH. The Lan CT2001A system was employed to perform discharge tests under various conditions. Each discharge cycle lasted for 10 minutes. Furthermore, measurements for open circuit potential (OCP) and power density were conducted using a CHI 760E electrochemical analyzer system.

**Figures**

**Figure S1.** SEM image of ZIF-8.

**Figure S2.** (a) SEM and (b) TEM images of GdCo-ZIF.

**Figure S3.** (a) SEM and (b) TEM images of Co-ZIF.

**Figure S4.** Nitrogen adsorption/desorption isotherm curves of (a) GdCo/NC; (b) Co/NC.

**Figure S5.** (a) SEM image; (b) TEM image; (c) EDX element mappings of Co/NC.

**Figure S6.** XPS survey spectrum of GdCo/NC.

**Figure S7.** Quantitative comparison of nitrogen configurations in GdCo/NC and Co/NC based on the fitted N 1s XPS spectra.

**Figure S8.** TEM images of (a) GdCo/NC and (b) Co/NC pyrolyzed at 800 ^o^C.

**Figure S9.** TEM images of (a) GdCo/NC and (b) Co/NC pyrolyzed at 1000 ^o^C.

**Figure S10.** LSV curves of GdCo/NC pyrolyzed at different temperatures.

**Figure S11.** TEM images of (a) GdCo/NC (Gd:Co=1:1); (b) GdCo/NC (Gd:Co=1:10).

**Figure S12.** XRD patterns of samples with different Gd contents.

**Figure S13.** LSV curves of Gd/NC and GdCo/NC.

**Figure S14.** CV curves under different scan rates for (a) GdCo/NC and (b) Co/NC.

**Figure S15.** (a) ECSA-normalized kinetic current density *J*_k_ ;(b) BET-normalized kinetic current density *J*_k_ of GdCo/NC and Co/NC.

**Figure S16.** Polarization curves of GdCo/NC at different rotation rates.

**Figure S17.** Koutecky-Levich plots of GdCo/NC.

**Figure S18.** TEM images of (a) GdCo/NC and (b) Co/NC after ORR performance.

**Figure S19.** XRD patterns of GdCo/NC and Co/NC after ORR performance.

**Figure S20.** High resolution Gd 4d of GdCo/NC before and after *i-t* test.

**Figure S21.** High resolution Co 2p of (a) GdCo/NC; (b) Co/NC before and after *i-t* test.

**Figure S22.** Three possible GdCo/NC models and the corresponding *E*_form_ after structural relaxation.

**Figure S23.** Electron localization function (ELF) maps of (a) Co/NC and (b) GdCo/NC.

**Figure S24.** Bader charge for (a) Co/NC and (b) GdCo/NC.

**Figure S25.** The optimized structure of various oxygen intermediates over Co/NC.

**Figure S26.** The optimized structure of various oxygen intermediates over GdCo/NC.

**Figure S27**. Spin density distribution of (a) Co/NC and (b) GdCo/NC. The isosurface level was set as 0.005 e Å^-3^.

**Figure S28.** LSV curves and Tafel plots of GdCo/NC, Co/NC and RuO_2_ at 0.1 M KOH.

**Figure S29.** OER polarization curves after 2000 cycles for GdCo/NC and Co/NC.

**Figure S30.** Free energy pathway of OER for GdCo/NC and Co/NC.

For Co/NC, *OH formation is identified as the RDS with a free-energy change of 1.67 eV, whereas the introduction of Gd shifts the RDS to *OH oxidation with a reduced free-energy requirement of 1.57 eV. These results indicate that the Gd incorporation regulates the adsorption and conversion energetics of oxygenated intermediates, thereby facilitating the OER process.

**Figure S31.** Enlarged Galvanostatic cycling of the rechargeable GdCo/NC-based ZAB at 5 mA cm^-2^ from (a) 0-5 h; (b) 425-430 h; (c) 775-780 h.

**Table S1.** Comparison of the ORR activity between GdCo/NC and other catalysts under basic conditions (0.1 M KOH) in literature.

| Catalysts | *E*_1/2_  (V *vs.*RHE) | Tafel Slope  (mV dec^-1^) | Ref. |
| --- | --- | --- | --- |
| GdCo/NC | 0.861 | 89.5 | This work |
| Co-LC/NC | 0.840 | 96.4 | ^[17]^ |
| CuFeCo-NC | 0.820 | 99.5 | ^[18]^ |
| Co_100%_-NC | 0.831 | 94.6 | ^[19]^ |
| Co@NC | 0.800 | 114.5 | ^[20]^ |
| CoBNPCF-1000 | 0.853 | 110.7 | ^[21]^ |
| Co,Fe@NCNF-800 | 0.840 | 154.0 | ^[22]^ |
| CeO_2_/BC700 | 0.600 | 96.0 | ^[23]^ |
| Ce-HPCNs | 0.831 | 91.0 | ^[24]^ |
| La/La_2_O_2_S | 0.820 | 93.0 | ^[25]^ |
| Ce-PGCN NS/Co_3_O_4_ | 0.700 | 137.0 | ^[26]^ |
| LaSA-NC | 0.790 | 33.6 | ^[27]^ |
| Pt/C+CeO_x_/C | 0.807 | 120.0 | ^[28]^ |
| Co_3_O_4_/CeO_2_ | 0.750 | 127.6 | ^[29]^ |
| LNO-F | 0.620 | 93.1 | ^[30]^ |
| Er_2_O_3_/CNF | 0.703 | 162.0 | ^[31]^ |

**Reference**

[1] D. San Roman, D. Krishnamurthy, R. Garg, H. Hafiz, M. Lamparski, N. T. Nuhfer, V. Meunier, V. Viswanathan; T. Cohen-Karni, "Engineering Three-Dimensional (3D) Out-of-Plane Graphene Edge Sites for Highly Selective Two-Electron Oxygen Reduction Electrocatalysis" *ACS Catalysis* **10**, (2020): 1993-2008.

[2] J. Hafner, "Ab-initio simulations of materials using VASP: Density-functional theory and beyond" *Journal of Computational Chemistry* **29**, (2008): 2044-2078.

[3] G. Kresse; D. Joubert, "From ultrasoft pseudopotentials to the projector augmented-wave method" *Physical Review B* **59**, (1999): 1758-1775.

[4] P. E. Blöchl, "Projector augmented-wave method" *Physical Review B* **50**, (1994): 17953-17979.

[5] J. P. Perdew, K. Burke; M. Ernzerhof, "Generalized Gradient Approximation Made Simple" *Physical Review Letters* **78**, (1997): 1396-1396.

[6] B. Hammer, L. B. Hansen; J. K. Nørskov, "Improved adsorption energetics within density-functional theory using revised Perdew-Burke-Ernzerhof functionals" *Physical Review B* **59**, (1999): 7413-7421.

[7] S. L. Dudarev, G. A. Botton, S. Y. Savrasov, C. J. Humphreys; A. P. Sutton, "Electron-energy-loss spectra and the structural stability of nickel oxide: An LSDA+U study" *Physical Review B* **57**, (1998): 1505-1509.

[8] X. Shi, S. L. Bernasek; A. Selloni, "Formation, Electronic Structure, and Defects of Ni Substituted Spinel Cobalt Oxide: a DFT+U Study" *The Journal of Physical Chemistry C* **120**, (2016): 14892-14898.

[9] G. Kresse; J. Furthmüller, "Efficiency of ab-initio total energy calculations for metals and semiconductors using a plane-wave basis set" *Computational Materials Science* **6**, (1996): 15-50.

[10] G. Kresse; J. Furthmüller, "Efficient iterative schemes for ab initio total-energy calculations using a plane-wave basis set" *Physical Review B* **54**, (1996): 11169-11186.

[11] Y. G. Zhang, G. B. Zhang; Y. Xu Wang, "First-principles study of the electronic structure and optical properties of Ce-doped ZnO" *Journal of Applied Physics* **109**, (2011): 063510.

[12] A. Jain, S. P. Ong, G. Hautier, W. Chen, W. D. Richards, S. Dacek, S. Cholia, D. Gunter, D. Skinner, G. Ceder; K. A. Persson, "Commentary: The Materials Project: A materials genome approach to accelerating materials innovation" *APL Materials* **1**, (2013): 011002.

[13] H. A. Hansen, J. Rossmeisl; J. K. Nørskov, "Surface Pourbaix diagrams and oxygen reduction activity of Pt, Ag and Ni(111) surfaces studied by DFT" *Physical Chemistry Chemical Physics* **10**, (2008): 3722-3730.

[14] X. Wang, J. Hu, T. Lu, H. Wang, D. Sun, Y. Tang, H. Li; G. Fu, "Importing Atomic Rare-Earth Sites to Activate Lattice Oxygen of Spinel Oxides for Electrocatalytic Oxygen Evolution" *Angewandte Chemie International Edition* **64**, (2025): e202415306.

[15] M. Li, X. Wang, K. Liu, Z. Zhu, H. Guo, M. Li, H. Du, D. Sun, H. Li, K. Huang, Y. Tang; G. Fu, "Ce-Induced Differentiated Regulation of Co Sites via Gradient Orbital Coupling for Bifunctional Water-Splitting Reactions" *Advanced Energy Materials* **13**, (2023): 2301162.

[16] J. K. Nørskov, J. Rossmeisl, A. Logadottir, L. Lindqvist, J. R. Kitchin, T. Bligaard; H. Jónsson, "Origin of the Overpotential for Oxygen Reduction at a Fuel-Cell Cathode" *The Journal of Physical Chemistry B* **108**, (2004): 17886-17892.

[17] X. Gao, Y. Li, Z. Zhang, H. Zhang; G. Li, "Architecting hydrangea-inspired nitrogen-doped hollow carbon with isolated Co atoms for superior oxygen reduction catalysis" *Journal of Materials Chemistry A* **13**, (2025): 14631-14645.

[18] H. Man, G. Chen, F. Wang, J. Ruan, Y. Liu, Y. Liu, F. Fang; R. Che, "Entropy Engineering Activates Cu-Fe Inertia Center From Prussian Blue Analogs With Micro-Strains for Oxygen Electrocatalysis in Zn-Air Batteries" *Carbon Energy* **7**, (2025): e693.

[19] Z. Li, W. Kang, J. Lin, R. Li, K. Qu, S. Zeng, L. Wang, F. Meng, H. Zhang; H. Li, "Single-Atom Co Meets Remote Fe for a Synergistic Boost in Oxygen Electrocatalysis" *Advanced Energy Materials* **15**, (2025): 2500617.

[20] C. Fan, X. Wang, X. Wu, Y. Chen, Z. Wang, M. Li, D. Sun, Y. Tang; G. Fu, "Neodymium-Evoked Valence Electronic Modulation to Balance Reversible Oxygen Electrocatalysis" *Advanced Energy Materials* **13**, (2023): 2203244.

[21] T. Fu, H. Xiao, Q. Liu, Y. Yu, Z. Che, Y. Zhang, A. Chen, M. Li; T. Liu, "Desirable Pore Connectivity Effects in Multiscale Interactive Cobalt Centers with Asymmetric B/N-Coordination Carbon for Promoting Zn-Air Batteries" *Advanced Science* **12**, (2025): e14619.

[22] S. Ren, P. Zhang, X. Tang, J. Tang, J. Zhang, L. Chen, H. Zheng, Z. Tu, Y. Chen, Z. Wan; S. H. Chan, "Modulating Electronic Structure of Atomic Iron Catalyst by Metallic Cobalt Cooperation for Boosting Oxygen Reduction/Evolution Reactions in Rechargeable and Flexible Zinc-Air Battery" *Carbon Energy* **8**, (2026): e70234.

[23] N. Bhuvanendran, S. Ravichandran, S. Kandasamy, W. Zhang, Q. Xu, L. Khotseng, T. Maiyalagan; H. Su, "Spindle-shaped CeO_2_/biochar carbon with oxygen-vacancy as an effective and highly durable electrocatalyst for oxygen reduction reaction" *International Journal of Hydrogen Energy* **46**, (2021): 2128-2142.

[24] W. Xia, J. Li, T. Wang, L. Song, H. Guo, H. Gong, C. Jiang, B. Gao; J. He, "The synergistic effect of Ceria and Co in N-doped leaf-like carbon nanosheets derived from a 2D MOF and their enhanced performance in the oxygen reduction reaction" *Chemical Communications* **54**, (2018): 1623-1626.

[25] Z. Yang, J. Yang, C. Yu, J. Bai, X. Xie, N. Jiang, B. Chen, S. Dong, M. Xiang; H. Qin, "Rare-Earth Lanthanum Tailoring Mott-Schottky Heterojunction by Sulfur Vacancy Modification as a Bifunctional Electrocatalyst for Zinc-Air Battery" *Small Structures* **4**, (2023): 2200267.

[26] P. Pakzad, M. Mehrpooya, F. Pourfayaz; M. R. Ganjali, "Electrochemical aspects of Co_3_O_4_ nanorods supported on the cerium doped porous graphitic carbon nitride nanosheets as an efficient supercapacitor electrode and oxygen reduction reaction electrocatalyst" *International Journal of Hydrogen Energy* **48**, (2023): 16294-16319.

[27] J. Yang, J. Cui, Y. Zhuang, J. Yu; L. Peng, "Rare-earth modification optimizes porous Fe-N-C catalysts to boost oxygen reduction reaction for Zn-air batteries" *Journal of Rare Earths* **44**, (2026): 569-576.

[28] A. Kostuch, E. Negro, G. Pagot, S. Zoladek, I. A. Rutkowska, O. Siamuk, A. Chmielnicka, V. Di Noto; P. J. Kulesza, "Importance of defective structure of ceria additive on selectivity and stability of carbon-supported low-Pt-content-catalysts during oxygen reduction" *Catalysis Today* **441**, (2024): 114889.

[29] S. Guo, J. Wang, Y. Sun, L. Peng; C. Li, "Interface engineering of Co_3_O_4_/CeO_2_ heterostructure in-situ embedded in Co/N‑doped carbon nanofibers integrating oxygen vacancies as effective oxygen cathode catalyst for Li-O_2_ battery" *Chemical Engineering Journal* **452**, (2023): 139317.

[30] T. Ren, L.-K. Zhao, X. Zhang, X.-W. Gao, H. Chen, Z. Liu, D. Yang; W.-B. Luo, "Optimizing LaNiO_3_ surface structure for an efficient oxygen reduction reaction" *Journal of Materials Chemistry A* **13**, (2025): 14737-14742.

[31] X. Wang, M. Li, P. Wang, D. Sun, L. Ding, H. Li, Y. Tang; G. Fu, "Spin-Selective Coupling in Mott-Schottky Er_2_O_3_-Co Boosts Electrocatalytic Oxygen Reduction" *Small Methods* **7**, (2023): 2300100.
